# Drinking with parents: Different measures, different associations with underage heavy drinking?

**DOI:** 10.1177/1455072517740235

**Published:** 2017-11-21

**Authors:** Hilde Pape, Elin K. Bye

**Affiliations:** Norwegian Institute of Public Health, Norway; Norwegian Institute of Public Health, Norway

**Keywords:** adolescents, binge drinking, drinking with parents, drunkenness, measurement, parenting

## Abstract

**Aims:**

Is drinking with parents (DWP) likely to curb or to encourage adolescent heavy drinking? The scant number of studies addressing this issue have arrived at contradictory conclusions, which may reflect that different measures of DWP have been used. We pursued the assumption, taking potential confounding related to parental alcohol-specific rule-setting and parenting style into account.

**Method:**

Data stem from the Norwegian 2015 ESPAD survey of 15–16 year olds. Drinking with parents at the last drinking event and the frequency of DWP in the past year were assessed among those who had consumed alcohol (*n* = 1374). Severe drunkenness and binge drinking in the past month were the outcomes. Parental covariates were accounted for in Poisson regression models.

**Results:**

One in five (21%) had been drinking with their parents the last time they consumed alcohol, and this DWP measure was strongly and inversely related to both drunkenness and binge drinking. Adolescents who reported no DWP episodes in the past year (61%) and those who reported 1–2 such episodes (30%) barely differed with respect to the two outcomes. More frequent DWP (9%) was significantly associated with an increased risk of heavy episodic drinking, but the statistical impact on severe drunkenness was no longer significant when adjusting for parental covariates.

**Conclusions:**

Different measures of DWP were related differently to adolescent heavy drinking, indicating that studies based on DWP at the last drinking event are biased in favour of the view that adolescents may “learn” sensible drinking by consuming alcohol with their parents.

According to the Norwegian health authorities, parents should not offer alcohol to their underage offspring in any context, as it promotes heavy and harmful consumption. Indeed, several studies have documented a positive association between the two (van der Vorst, 2012; Ward & Snow, 2010; Yap, Cheong, Zaravinos-Tsakos, Lubman, & Jorm, 2017). Moreover, the widespread downward trend in adolescent drinking in the 2000s (de Looze et al., 2015; Kraus, Guttormsson, Leifman, Arpa, & Molinaro, 2016) has coincided with substantial decreases in parents’ supply of alcohol and more restrictive alcohol-related parenting in various countries (de Looze et al., 2014; Hallgren, Leifman, & Andréasson, 2012; Kelly et al., 2016; Raitasalo & Holmila, 2016). However, the evidence is insufficient to conclude that parental supply of alcohol is a causal factor (Sharmin et al., 2017), and some studies have in fact revealed that it is related *inversely* to risky use of alcohol by youth (Kaynak, Winters, Cacciola, Kirby, & Arria, 2014).

Obviously, the term “parental provision of alcohol” may refer to different phenomena. For instance, parents may offer sips or whole drinks, they may drink with their children in a supervised family setting or provide alcohol for partying with peers, and their supply may be confined to exceptional occasions or may occur more regularly. Few studies have distinguished between different forms and aspects of parental provision of alcohol, yet there is evidence to suggest that supply for unsupervised drinking is particularly risky (Gilligan, Kypri, Johnson, Lynagh, & Love, 2012).

Our study of Norwegian youth focuses specifically on underage drinking with parents (DWP). The potential implications of DWP on adolescents’ drinking behaviour more generally have long been surrounded by controversy (McMorris, Catalano, Kim, Toumbourou, & Hemphill, 2011), and scarce research has addressed the issue. Some scholars have vigorously warned against DWP, claiming that it conveys parental approval of alcohol use, which has been found to increase young people’s risk of heavy consumption (Ryan, Jorm, & Lubman, 2010; van der Vorst, 2012; Ward & Snow, 2010). Others have argued in favour of a harm-reduction strategy, claiming that youngsters learn to drink sensibly by consuming limited amounts of alcohol with their parents (Foxcroft & Lowe, 1997; Green, Macintyre, West, & Ecob, 1991; Peele, 2007). The assumption is that such drinking practices yield a protective learning effect that generalizes to unsupervised alcohol use in other contexts. Studies from Australia and the United States indicate that quite a few parents share this view (Gilligan et al., 2012; Jackson, Henriksen, & Dickinson, 1999; Jones, Andrews, & Berry, 2016).

The study by Foley, Altman, DuRant, and Wolfson (2004) has been cited frequently both in the popular press and in the research literature as evidence in support of the harm-reduction strategy. The researchers applied a measure of DWP at the last drinking event, and found that it was related inversely to extensive use of alcohol by youth. The conclusion was that “drinking with parents […] appears to have a protective effect on general drinking trends” (Foley et al., 2004, p. e22). A few other studies have shown similar results (Mayer, Forster, Murray, & Wagenaar, 1998; Reboussin, Song, & Wolfson, 2012; Song, Smiler, Wagoner, & Wolfson, 2012). Without exception, these studies also assessed DWP by asking about the social context of the last drinking event.

However, the “last event” measure of DWP has recently been called into question (Blanchette & Heeren, 2013; Jones, 2016). The intake of alcohol is far lower when adolescents drink with their parents rather than with their peers (Foley et al., 2004; Kask & Markina, 2014; Mayer et al., 1998), and the probability that DWP occurred at the last drinking event is likely to be elevated among those who have experienced DWP but who rarely drink in a peer context. Conversely, adolescents who frequently get drunk are probably less likely to report DWP at the last drinking event, simply because excessive drinking typically occurs when the parents are out of sight. Blanchette and Heeren (2013) thus argue that studies based on a “last event” measure of DWP are likely to be biased in favour of the harm-reduction approach.

The international research literature seems to include only a couple of studies that have assessed DWP differently, and they do not lend support to the view that drinking with parents may serve a harm-reducing function. Pape, Rossow, and Storvoll (2015) used a frequency measure of DWP in a previous study of Norwegian youth, and found that it correlated positively with heavy episodic drinking. Moreover, a longitudinal study by Degenhardt et al. (2015) showed that youth who reported recurrent (3+ times) drinking in any context, including drinking with their parents in a family setting, had an increased likelihood of later risky consumption.

## The present study

In contrast to previous research, our study included two different measures of drinking with parents: DWP at the last drinking event, and the frequency of DWP in the past year. Hence, we had a unique opportunity to investigate whether these measures correlated differently with adolescent heavy drinking. The dataset also included indicators for parenting style, which were taken into account. Pape et al. (2015) found an inverse association between the frequency of drinking with parents and indicators for parenting quality, yet few other studies have assessed such parental correlates of DWP. There is, however, solid evidence that both general and alcohol-related parenting impact on the risk of excessive use of alcohol by youth (Ahlström, 2002; Kaynak et al., 2014; Ryan et al., 2010; van der Vorst, 2012; Ward & Snow, 2010; Yap et al., 2017). For instance, strict rules against unsupervised drinking and high levels of parental care and support have been identified as protective factors.

## Method

### Sample and design

The European School Survey Project on Alcohol and Other Drugs (ESPAD) is conducted every fourth year and includes samples of 10th graders (15–16 year olds) from almost all European countries (Kraus et al., 2016). We used data from the 2015 Norwegian ESPAD survey. School classes were randomly selected after stratification by school size, region, and geography to yield a nationally representative sample of students. Attrition at the school level was quite high, so the response rate was moderate when non-participating schools were taken into account (53%), while it was high when they were excluded (90%) (*N* = 2805). The net sample covered all 19 counties in Norway, and the geographical distribution of the respondents was fairly proportional to the size of the youth population in the four major regions in the country. Data were collected using anonymous self-report questionnaires that were administered during ordinary school hours.

Of the respondents, 11% reported that neither of their parents had consumed alcohol in the past year, while 48% had not been drinking in the past year themselves. These respondents had ipso facto not experienced any DWP episodes in the same period, and were therefore excluded. We also excluded about 1% of the respondents in the remaining sample due to missing data on one or both DWP measures. The final study sample comprised 1374 adolescents, of whom 52% were boys.

### Measures

Items on DWP and parental alcohol-related permissiveness were included only in the Norwegian survey, whereas all other items stem from the standard ESPAD questionnaire (Kraus et al., 2016).

#### Drinking outcomes

We applied two dichotomous measures of heavy episodic drinking: severe drunkenness and binge drinking in the past 30 days. The former was operationalised as having been “intoxicated from drinking alcoholic beverages, for example staggered when walking, not being able to speak properly, throwing up, or not remembering what happened”. Binge drinking was assessed through a question about intake of five or more units of alcohol on one single drinking occasion.

#### Drinking with parents (DWP) was assessed through two questions

(i) Did you drink with your parents (one or both) the last time you drank alcohol? (ii) How many times in the past 12 months have you been drinking with your parents (one or both)? The response options for the latter question were “no times”, “1–2 times”, “3–5 times”, and “6 or more times”. Very few (3%) had experienced DWP 6+ times, and we thus applied a trichotomous measure with “3+ times” as the highest frequency category. When referring to drinking with parents at the last drinking event, we will occasionally use the term “DWP last time”.

#### Parenting factors

We assessed *alcohol-related parental permissiveness* through an item about parents’ rule-setting with respect to drinking. Adolescents who were allowed to drink without getting drunk and those who reported no parental restrictions were merged and contrasted with those who reported strict parental rules against alcohol use. Moreover, the adolescents reported the extent to which six statements about their parents corresponded with their impressions or experiences on a scale ranging from 1 (hardly ever) to 5 (almost always). An exploratory factor analysis of the items resulted in a readily interpretable three-factor solution: (i) *Parental knowledge* (“My parent(s) know who I’m with in the evenings”, and “My parent(s) know where I am in the evenings”), (ii) *Parental care* (“I can easily get warmth and caring from my mother and/or father” and “I can easily get emotional support from my mother and/or father”), and (iii) *Parental rule-setting* (“My parent(s) set definite rules about what I can do at home” and “My parent(s) set definite rules about what I can do outside the home”). The items for each of the parenting factors were added up and averaged. The Spearman–Brown coefficients for the three indices ranged from 0.82 to 0.92, implying satisfactory reliability (Eisinga, Grotenhuis, & Pelzer, 2013).

### Statistical analyses

Variations in proportions were assessed using cross-tabulations with χ^2^-test while ANOVA with F-test was used to examine differences between means. We also applied Poisson regression to estimate relative influence of DWP on heavy episodic drinking. This estimation procedure is more robust to omitted variables than logistic regression (Zou, 2004), and relative risks are also easier to interpret than odds ratios. Because the sampling was clustered by school, we used robust clustered standard errors with school as cluster variable (Williams, 2000). Data were analysed using STATA (version 14).

## Results

Table 1 shows that one in five adolescents (21%) had consumed alcohol with their parents at their last drinking episode. Moreover, 30% reported 1–2 DWP episodes in the past year, and 9% had experienced DWP more frequently (3+ times). The prevalence of severe drunkenness in the past month was 16%, while the percentage reporting past month binge drinking was 40%. Neither of the DWP measures varied by gender, nor were there any statistically significant differences between boys and girls with respect to the two drinking outcomes. Hence, none of the subsequent analyses were conducted separately for boys and girls, nor did we adjust for the respondents’ gender in multivariate analyses.

**Table 1.** Drinking with parents (DWP) and heavy episodic drinking in the full sample and by gender – percentages.

	All	Boys	Girls	*p*
DWP last time	20.8	21.9	19.6	0.300
Frequency of DWP				
1–2 times	30.2	30.7	29.6	0.876
3+ times	8.8	8.9	8.7	
Severe drunkenness	16.3	16.8	15.7	0.580
Binge drinking	39.8	41.0	38.6	0.376
Lowest *N*	1295	671	624	–

The upper part of Table 2 shows that DWP at the last drinking event was strongly and inversely related to both outcome measures of heavy episodic drinking. For instance, the prevalence of past month drunkenness was nearly seven times higher among those who did not report “DWP last time” than among those who did (20% versus 3%). The associations between the past year frequency of DWP and the two outcomes went in the opposite direction. That is, the proportion reporting severe drunkenness was substantially elevated among those who had experienced 3+ DWP episodes, as was the proportion reporting binge drinking. There were, however, only small differences between the “no DWP” group and those who had consumed alcohol with parents 1–2 times.

**Table 2.** Variations in severe drunkenness, binge drinking and parenting factors by drinking with parents (DWP) at the last drinking event and the frequency of DWP in the past year – percentages and means (SD).

	DWP last time			Frequency of DWP			
	No	Yes	*p*	0	1–2	3+	*p*
Drinking outcomes:							
Severe drunkenness (%)	20.0	3.2	< 0.001	15.8	14.2	28.7	< 0.001
Binge drinking (%)	46.7	14.5	< 0.001	39.1	36.3	59.3	< 0.001
Parenting factors:							
Alcohol-related parental permissiveness (%)	21.4	25.9	0.107	16.0	28.2	46.7	< 0.001
Parental knowledge^1^	4.1 (0.85)	4.4 (0.76)	< 0.001	4.2 (0.80)	4.3 (0.84)	3.9 (1.06)	< 0.001
Parental care^1^	4.2 (0.93)	4.3 (0.83)	0.136	4.3 (0.92)	4.3 (0.85)	3.9 (1.02)	< 0.001
Parental rule-setting^1^	3.4 (1.02)	3.3 (1.03)	0.111	3.4 (1.02)	3.3 (0.98)	2.9 (1.07)	< 0.001
*Lowest N*	1049	276	–	809	409	115	–

^1^Scale range: 1 (low)–5 (high level).

The lower part of Table 2 shows that parental knowledge was related positively to “DWP last time”. None of the other parenting factors correlated significantly with this measure of drinking with parents. In contrast, there was a consistent pattern of significant associations with the past year frequency of DWP. That is, the proportion reporting alcohol-related parental permissiveness was lowest among adolescents who reported no such drinking episodes and highest among those reporting DWP 3+ times. The latter group also reported far lower levels of parental knowledge, care, and rule-setting than others. As regards all the three indicators of general parenting style, the results for the “no DWP” group and for those who had experienced DWP 1–2 times showed little variation.

As one might expect from the results in Table 2, the inverse statistical impact of DWP at the last drinking event on the two outcomes barely changed when we accounted for the parenting factors (Table 3). The estimate implies that those who reported “DWP last time” had an expected risk of severe drunkenness that was 82% lower (Adj RR = 0.18) than those who did not, while their risk of binge drinking was 67% lower (Adj RR = 0.33). As regards the frequency of DWP, there were no indications that 1–2 such drinking episodes made any difference, mirroring the results in Table 2. Moreover, the relative risk of more frequent DWP (3+) on severe drunkenness was no longer statistically significant when adjusting for the parenting factors. The impact of relatively frequent DWP on binge drinking also declined when these factors were accounted for, but not below the level of statistical significance. Specifically, the adjusted risk of consuming 5+ alcohol units on one single occasion was 24% higher (Adj RR = 1.24) among adolescents who reported 3+ DWP episodes as compared to those who reported no DWP episodes.

**Table 3.** The statistical impact of drinking with parents (DWP) at the last drinking event and the frequency of DWP in the past year on severe drunkenness and binge drinking in the past month – crude and adjusted relative risks (RR)^1^ with 95% confidence intervals (CI).

	Severe drunkenness		Binge drinking	
	Crude RR (95% CI)	Adj RR^1^ (95% CI)	Crude RR (95% CI)	Adj RR^1^ (95% CI)
DWP last time^2^	0.16** (0.08–0.30)	0.18** (0.09–0.33)	0.31** (0.23–0.42)	0.33** (0.25–0.44)
Frequency of DWP^2^				
1–2 times	0.90 (0.65–1.24)	0.88 (0.64–1.20)	0.93 (0.78–1.10)	0.90 (0.77–1.06)
3+ times	1.81* (1.24–2.65)	1.33 (0.91–1.95)	1.52** (1.26–1.82)	1.24** (1.03–1.50)

^1^Adjusted estimates are controlled for parental knowledge, parental care, parental rule-setting, and alcohol-related parental permissiveness.

^2^Ref: No DWP.

* *p* < 0.01, ** *p* < 0.001.

## Discussion

Is drinking with parents (DWP) associated with an increased or a decreased risk of adolescent heavy drinking, or is there no association between the two? Our study expanded the meagre and inconsistent body of research that addresses this question. We used data on DWP at the last drinking event and the frequency of DWP in the past year, and found that the two measures were related differently to heavy episodic drinking. Moreover, we applied various indicators for parenting style and revealed that their association with each of the two DWP measures also differed in terms of both strength and direction.

In line with some previous studies (Foley et al., 2004; Mayer et al., 1998; Reboussin et al., 2012; Song et al., 2012), DWP at the last drinking event was associated strongly and inversely with past-month drunkenness and binge drinking. The decreased relative risk of “DWP last time” remained highly significant and became only negligibly weaker when accounting for alcohol-related parental permissiveness and the indicators for general parenting style. Thus, the parental factors that we gauged were generally unrelated to “DWP last time”, yet there was one notable exception: the level of parental knowledge correlated positively with this measure of drinking with parents. Other studies provide evidence that parents’ knowledge of their children’s social life and whereabouts is indicative of a high-quality parent–child relationship (Crouter & Head, 2002; Stattin & Kerr, 2000), which recently was identified as one of the most important parental factors that protects against excessive adolescent drinking (Yap et al., 2017).

We found no indications of a potential harm-reducing effect when applying the past-year frequency measure of DWP rather than “DWP last time”. That is, adolescents who had experienced no such drinking episodes and those who reported DWP 1–2 times were about equally likely to engage in heavy episodic drinking. As regards parents’ alcohol-related permissiveness and the three indicators of parenting style (parental knowledge, care, and rule-setting), the results for the two groups also showed little variation. More frequent DWP (3+ times) was related positively to the drinking outcomes – which corroborates the results of other studies that have applied frequency measures of consuming alcohol with parents (Degenhardt et al., 2015; Pape et al., 2015). Relatively frequent DWP also correlated significantly with all the parenting factors, indicating that such drinking practices were embedded in a pattern of less optimal parenting. The relative risk of 3+ DWP episodes was attenuated when adjusting for the parental factors, and the impact on severe drunkenness was no longer statistically significant. These results corroborate those reported by Pape et al. (2015) in a previous study of Norwegian teenagers.

When interpreting the findings of our study, we need to take the cultural context into account. Compared to other parts of Europe, adolescents in the Nordic countries are generally less likely to consume alcohol with their parents, and the prevalence of DWP is particularly low in Norway (Kask & Markina, 2014). Southern European countries are located at the opposite end of the scale. Indeed, adolescent drinking in a family context has been described as customary in wine-producing countries such as Italy and France (Beccaria & Sande, 2003; Heath, 1995), and the association between relatively frequent DWP and heavy episodic drinking may not be present in those countries. The association with suboptimal parenting may also be culture-specific. Negligible gender differences in the prevalence of drinking with parents have, however, been documented in both northern, western and southern parts of Europe (Kask & Markina, 2014).

Our findings clearly supported the critical reflections by Blanchette and Heeren (2013), who suspected research based on DWP at the last drinking event to be biased in favour of the view that adolescents learn sensible drinking by consuming alcohol with their parents. Applying data on the last drinking event may also be problematic in other contexts. For instance, such data are likely to produce erroneous estimates of the total alcohol consumption because the intake at the last drinking event may not be representative of individuals’ typical volume of drinking (Østhus & Brunborg, 2015). Correspondingly, if one assesses the occurrence of DWP in a given period, rather than DWP at the last drinking event, one may to a greater extent capture adolescent high-risk drinkers who only exceptionally consume alcohol with their parents.

### Methodological considerations

The attrition at the school level was quite substantial, but it seems unlikely that it was biased with respect to DWP or heavy episodic drinking. Moreover, the response rate at the participating schools was high, and the net sample included students from all counties in Norway. It is likely that our study was fairly representative of the population of Norwegian 10th graders.

However, the cross-sectional study design implies that the temporal order of DWP and the drinking outcomes could not be determined. Although we used measures of heavy episodic drinking in the past month while there was a one-year time frame for consuming alcohol with parents, one cannot disregard the possibility that adolescent heavy drinking may be predictive of relatively frequent DWP (3+ times).

It is, however, highly unlikely that frequent DWP correlated with the outcomes because a sizable proportion of the adolescents drank excessively when they consumed alcohol with their parents. Thus, as noted, the intake of alcohol is typically low when adolescents drink in such a social context (Foley et al., 2004; Kask & Markina, 2014; Mayer et al., 1998). It is still possible that a small subgroup tends to drink quite a lot when they consume alcohol with their parents, and a quantity/frequecy measure of DWP would have been more optimal than the crude frequency measure that we applied.

All the data in our study were adolescent-reported, implying measurement errors that reduce the precision of our estimates. On the other hand, there is evidence to suggest that parents may be reluctant to provide truthful information about their alcohol-related rules and practices (Friese, Grube, Moore, & Jennings, 2012; Kypri, Dean, & Stojanovski, 2007). Compared to adolescents’ reports, parents tend to portray themselves as more restrictive and politically correct (Livingston, Testa, Hoffman, & Windle, 2010; Varvil-Weld, Turrisi, Scaglione, Mallett, & Ray, 2013). There are also indications that adolescent-reported measures on parenting practices are more predictive of the adolescents’ drinking behaviour than are parent-reported measures (Cohen & Rice, 1997; Cottrell et al., 2003; Latendresse et al., 2009).

## Concluding remarks and suggestions for future research

This study explains why previous research on the association between DWP and underage heavy drinking has arrived at contradictory conclusions. The results clearly indicated that studies based on DWP at the last drinking event are biased in favour of the hypothesised harm-reduction effect of DWP, and that the *frequency* of DWP matters. Specifically, frequent – unlike infrequent – DWP was associated with an increased risk of heavy episodic drinking, which in part seemed to reflect variations in parenting quality. Our study thus tentatively suggests that DWP per se is not necessarily risky, and that parent-targeted measures against extensive use of alcohol by youth should address a wide variety of parenting skills and practices.

It is unknown whether our finding of an association between relatively frequent DWP and heavy episodic drinking reflects a causal relationship. Future research should assess the potential impact of DWP longitudinally, applying quantity/frequency measures of such drinking events and taking a broad range of parenting factors and other potential confounders into account. Moreover, scarce research has examined the nature and the situational characteristics of the occasions when underage youth and their parents consume alcohol together. Why some parents drink in this context and how much they consume when accompanied by their children are also issues that future research should address. A final suggestion is to conduct comparative cross-cultural research on the potential impact of DWP on adolescent drinking behaviour.

## References

[bibr1-1455072517740235] Ahlström S. (2002). Family practices and adolescent use of legal and illegal drugs: A review. Nordic Studies on Alcohol and Drugs, 19, 76–82.

[bibr2-1455072517740235] Beccaria F. Sande A. (2003). Drinking games and rite of life projects a social comparison of the meaning and functions of young people’s use of alcohol during the rite of passage to adulthood in Italy and Norway. Young, 11(2), 99–119.

[bibr3-1455072517740235] Blanchette J. Heeren T. (2013). Methodology may exaggerate a beneficial effect of drinking with parents. Journal of Studies on Alcohol and Drugs, 74(2), 353. 23384384 10.15288/jsad.2013.74.353

[bibr4-1455072517740235] Cohen D. A. Rice J. (1997). Parenting styles, adolescent substance use, and academic achievement. Journal of Drug Education, 27(2), 199–211. 9270213 10.2190/QPQQ-6Q1G-UF7D-5UTJ

[bibr5-1455072517740235] Cottrell L. Li X. Harris C. D’Alessandri D. Atkins M. Richardson B. Stanton B. (2003). Parent and adolescent perceptions of parental monitoring and adolescent risk involvement. Parenting: Science and Practice, 3(3), 179–195.

[bibr6-1455072517740235] Crouter A. C. Head M. R. (2002). Parental monitoring and knowledge of children. Handbook of Parenting, 3, 461–483.

[bibr7-1455072517740235] de Looze M. Raaijmakers Q. Ter Bogt T. Bendtsen P. Farhat T. Ferreira M.…Pförtner T.-K (2015). Decreases in adolescent weekly alcohol use in Europe and North America: Evidence from 28 countries from 2002 to 2010. The European Journal of Public Health, 25(suppl 2), 69–72. 25805792 10.1093/eurpub/ckv031 PMC4408543

[bibr8-1455072517740235] de Looze M. Vermeulen-Smit E. Ter Bogt T. F. van Dorsselaer S. A. Verdurmen J. Schulten I.…Vollebergh W. A. (2014). Trends in alcohol-specific parenting practices and adolescent alcohol use between 2007 and 2011 in the Netherlands. International Journal of Drug Policy, 25(1), 133–141. 24209833 10.1016/j.drugpo.2013.09.007

[bibr9-1455072517740235] Degenhardt L. Romaniuk H. Coffey C. Hall W. D. Swift W. Carlin J. B.…Patton G. C. (2015). Does the social context of early alcohol use affect risky drinking in adolescents? Prospective cohort study. BMC Public Health, 15(1), 1. 26572739 10.1186/s12889-015-2443-5 PMC4647618

[bibr10-1455072517740235] Eisinga R. Grotenhuis Mt. Pelzer B. (2013). The reliability of a two-item scale: Pearson, Cronbach, or Spearman-Brown? International Journal of Public Health, 58(4), 637–642. 23089674 10.1007/s00038-012-0416-3

[bibr11-1455072517740235] Foley K. L. Altman D. DuRant R. H. Wolfson M. (2004). Adults’ approval and adolescents’ alcohol use. Journal of Adolescent Health, 35(4), e17–e26. 15830441

[bibr12-1455072517740235] Foxcroft D. R. Lowe G. (1997). Adolescents’ alcohol use and misuse: The socializing influence of perceived family life. Drugs: Education, Prevention, and Policy, 4(3), 215–229.

[bibr13-1455072517740235] Friese B. Grube J. W. Moore R. S. Jennings V. K. (2012). Parents’ rules about underage drinking: A qualitative study of why parents let teens drink. Journal of Drug Education, 42(4), 379–391. 25031481 10.2190/DE.42.4.a PMC4095821

[bibr14-1455072517740235] Gilligan C. Kypri K. Johnson N. Lynagh M. Love S. (2012). Parental supply of alcohol and adolescent risky drinking. Drug and Alcohol Review, 31(6), 754–762. 22340514 10.1111/j.1465-3362.2012.00418.x

[bibr15-1455072517740235] Green G. Macintyre S. West P. Ecob R. (1991). Like parent like child? Associations between drinking and smoking behaviour of parents and their children. British Journal of Addiction, 86(6), 745–758. 1878624 10.1111/j.1360-0443.1991.tb03100.x

[bibr16-1455072517740235] Hallgren M. Leifman H. Andréasson S. (2012). Drinking less but greater harm: Could polarized drinking habits explain the divergence between alcohol consumption and harms among youth? Alcohol and Alcoholism, 47(5), 581–590. 22763231 10.1093/alcalc/ags071

[bibr17-1455072517740235] Heath D. B. (1995). International handbook on alcohol and culture. Westport, CT: Greenwood Press.

[bibr18-1455072517740235] Jackson C. Henriksen L. Dickinson D. (1999). Alcohol-specific socialization, parenting behaviors and alcohol use by children. Journal of Studies on Alcohol and Drugs, 60(3), 362. 10.15288/jsa.1999.60.362 10371264

[bibr19-1455072517740235] Jones S. C. (2016). Parental provision of alcohol: A TPB-framed review of the literature. Health Promotion International, 31(3), 562–571. 25908595 10.1093/heapro/dav028

[bibr20-1455072517740235] Jones S. C. Andrews K. Berry N. (2016). Lost in translation: A focus group study of parents’ and adolescents’ interpretations of underage drinking and parental supply. BMC Public Health, 16(1), 561. 27411789 10.1186/s12889-016-3218-3 PMC4944521

[bibr21-1455072517740235] Kask K. Markina A. (2014). With whom did you drink last time? An analysis of adolescents’ alcohol use. Annual Research & Review in Biology, 4(1), 174.

[bibr22-1455072517740235] Kaynak Ö. Winters K. C. Cacciola J. Kirby K. C. Arria A. M. (2014). Providing alcohol for underage youth: What messages should we be sending parents? Journal of Studies on Alcohol and Drugs, 75(4), 590–605. 24988258 10.15288/jsad.2014.75.590 PMC4108600

[bibr23-1455072517740235] Kelly A. B. Chan G. C. Weier M. Quinn C. Gullo M. J. Connor J. P. Hall W. D. (2016). Parental supply of alcohol to Australian minors: An analysis of six nationally representative surveys spanning 15 years. BMC Public Health, 16(1), 325. 27074975 10.1186/s12889-016-3004-2 PMC4831148

[bibr24-1455072517740235] Kraus L. Guttormsson U. Leifman H. Arpa S. Molinaro S. (2016). ESPAD Report 2015: Results from the European School Survey Project on Alcohol and Other Drugs. Luxembourg: Publications Office of the European Union.

[bibr25-1455072517740235] Kypri K. Dean J. I. Stojanovski E. (2007). Parent attitudes on the supply of alcohol to minors. Drug and Alcohol Review, 26(1), 41–47. 17364835 10.1080/09595230601037018

[bibr26-1455072517740235] Latendresse S. J. Rose R. J. Viken R. J. Pulkkinen L. Kaprio J. Dick D. M. (2009). Parental socialization and adolescents’ alcohol use behaviors: Predictive disparities in parents’ versus adolescents’ perceptions of the parenting environment. Journal of Clinical Child & Adolescent Psychology, 38(2), 232–244. 19283601 10.1080/15374410802698404 PMC3641770

[bibr27-1455072517740235] Livingston J. A. Testa M. Hoffman J. H. Windle M. (2010). Can parents prevent heavy episodic drinking by allowing teens to drink at home? Addictive Behaviors, 35(12), 1105–1112. 20805017 10.1016/j.addbeh.2010.08.005 PMC2942998

[bibr28-1455072517740235] Mayer R. R. Forster J. L. Murray D. M. Wagenaar A. C. (1998). Social settings and situations of underage drinking. Journal of Studies on Alcohol and Drugs, 59(2), 207. 10.15288/jsa.1998.59.207 9500308

[bibr29-1455072517740235] McMorris B. J. Catalano R. F. Kim M. J. Toumbourou J. W. Hemphill S. A. (2011). Influence of family factors and supervised alcohol use on adolescent alcohol use and harms: Similarities between youth in different alcohol policy contexts. Journal of Studies on Alcohol and Drugs, 72(3), 418. 21513678 10.15288/jsad.2011.72.418 PMC3084357

[bibr30-1455072517740235] Østhus S. Brunborg G. S. (2015). Why the “last drinking occasion” approach to measuring alcohol consumption should be avoided. Drug and Alcohol Review, 34(5), 549–558. 26121105 10.1111/dar.12293

[bibr31-1455072517740235] Pape H. Rossow I. Storvoll E. (2015). Is drinking with parents associated with high-risk drinking among adolescents? European Addiction Research, 21(6), 291–299. 26022605 10.1159/000381673

[bibr32-1455072517740235] Peele S. (2007). Addiction proof your child: A realistic approach to preventing drug, alcohol, and other dependencies. New York, NY: Random House/Three Rivers Press.

[bibr33-1455072517740235] Raitasalo K. Holmila M. (2016). Practices in alcohol education among Finnish parents: Have there been changes between 2006 and 2012? Drugs: Education, Prevention and Policy. Advance online publication.

[bibr34-1455072517740235] Reboussin B. A. Song E.-Y. Wolfson M. (2012). Social influences on the clustering of underage risky drinking and its consequences in communities. Journal of Studies on Alcohol and Drugs, 73(6), 890. 23036206 10.15288/jsad.2012.73.890 PMC3469043

[bibr35-1455072517740235] Ryan S. M. Jorm A. F. Lubman D. I. (2010). Parenting factors associated with reduced adolescent alcohol use: A systematic review of longitudinal studies. Australian and New Zealand Journal of Psychiatry, 44(9), 774–783. 20815663 10.1080/00048674.2010.501759

[bibr36-1455072517740235] Sharmin S. Kypri K. Khanam M. Wadolowski M. Bruno R. Mattick R. P. (2017). Parental supply of alcohol in childhood and risky drinking in adolescence: Systematic review and meta-analysis. International Journal of Environmental Research and Public Health, 14(3), 287. 10.3390/ijerph14030287 PMC5369123 28282955

[bibr37-1455072517740235] Song E.-Y. Smiler A. P. Wagoner K. G. Wolfson M. (2012). Everyone says it’s OK: Adolescents’ perceptions of peer, parent, and community alcohol norms, alcohol consumption, and alcohol-related consequences. Substance Use & Misuse, 47(1), 86–98. 22216994 10.3109/10826084.2011.629704

[bibr38-1455072517740235] Stattin H. Kerr M. (2000). Parental monitoring: A reinterpretation. Child Development, 71(4), 1072–1085. 11016567 10.1111/1467-8624.00210

[bibr39-1455072517740235] van der Vorst H. (2012). The role of parents in adolescents’ alcohol use In Verster K. B. Galanter M. Conrod P. (Eds.), Drug abuse and addiction in medical illness (pp. 497–504). New York, NY: Springer.

[bibr40-1455072517740235] Varvil-Weld L. Turrisi R. Scaglione N. Mallett K. A. Ray A. E. (2013). Parents’ and students’ reports of parenting: Which are more reliably associated with college student drinking? Addictive Behaviors, 38(3), 1699–1703. 23254221 10.1016/j.addbeh.2012.09.017 PMC3558596

[bibr41-1455072517740235] Ward B. Snow P. (2010). Supporting parents to reduce the misuse of alcohol by young people. Drugs: Education, Prevention and Policy, 17(6), 718–731.

[bibr42-1455072517740235] Williams R. L. (2000). A note on robust variance estimation for cluster-correlated data. Biometrics, 56, 645–646. doi:10.1111/j.0006-341X.2000.00645.x 10877330 10.1111/j.0006-341x.2000.00645.x

[bibr43-1455072517740235] Yap M. B. Cheong T. W. Zaravinos-Tsakos F. Lubman D. I. Jorm A. F. (2017). Modifiable parenting factors associated with adolescent alcohol misuse: A systematic review and meta-analysis of longitudinal studies. Addiction, 112(7), 1142–1162. 28178373 10.1111/add.13785

[bibr44-1455072517740235] Zou G. (2004). A modified poisson regression approach to prospective studies with binary data. American Journal of Epidemiology, 159(7), 702–706. 15033648 10.1093/aje/kwh090

